# The Prevalence and Association of Non-metric Dental Traits With Dentoskeletal Malocclusion and ABO Blood Groups in the Maharashtrian Population

**DOI:** 10.7759/cureus.59853

**Published:** 2024-05-07

**Authors:** Lalitkumar Gade, Priyanka P Kamble, Abdul Suban A Kanna, Kishor Patil, Ketan Saraf, Bhanu P Singh

**Affiliations:** 1 Department of Oral Pathology, Sau Mathurabai Bhausaheb Thorat (SMBT) Dental College and Hospital, Sangamner, IND; 2 Department of Oral Pathology, Jawahar Medical Foundation (JMF) Annasaheb Chudaman Patil Memorial (ACPM) Dental College, Dhule, IND; 3 Department of Orthodontics, Sri Ramakrishna Dental College and Hospital, Coimbatore, IND; 4 Department of Orthodontics, Kothiwal Dental College and Research Centre, Moradabad, IND

**Keywords:** association, forensic dentistry, malocclusion, abo blood group, traits

## Abstract

Introduction

Non-metric dental traits (NMDTs) are a fundamental data source in forensic dentistry. Nevertheless, the insufficiency of data regarding the occurrence of these traits has instigated the present research endeavor aimed at ascertaining the prevalence, sexual dimorphism, and extent of inter-trait correlations within the Maharashtrian population of India. The secondary objective was to determine the correlations between NMDTs, dentoskeletal malocclusion, and ABO blood groups.

Materials and methods

This prospective, observational study included 528 individuals aged 18-30 years with dentoskeletal Class I, II, and III malocclusions. NMDTs such as the presence of Cusp of Carabelli (CoC) on the upper first molars, hypocone on the upper second molars, and tri- or bicuspid lower second premolars were observed on the dental casts of all individuals. The dental relationship was assessed clinically according to Angle’s system for the classification of malocclusion. The skeletal relationship was assessed using lateral cephalograms of the individuals. ABO blood groups were obtained from their medical records. The Chi-square test of independence was used to assess the associations between various variables. The correlation between each measurement was determined using Spearman’s correlation test. Multivariate analysis enabled the identification of parameters that exhibited independent associations with NMDTs. A multinomial logistic regression model was constructed using NMDTs as the outcome variable.

Results

The mean age of males was 20.82 ± 1.71 years and 21.15 ± 1.76 years was in females. NMDTs were predominantly seen in females (n=394, 75%), with Class II dentoskeletal malocclusion (n=265, 50%) and B blood group ((n=199, 38%). All traits showed bilateral predominance. A statistically significant association was found between CoC, dentoskeletal malocclusion, hypocone, and tricuspid lower second premolars (p <0.05). All NMDTs showed a negative correlation with sex, a positive correlation between age and the presence of hypocones and CoC, a negative correlation between age and tricuspid lower second premolars, a strong positive correlation with dentoskeletal malocclusion, and a weak positive correlation with ABO blood groups. Multinomial logistic regression model analysis revealed that none of the independent variables were statistically significant predictors of the presence of CoC and tricuspid lower second premolars, while dentoskeletal malocclusion and sex were significant predictors of the presence of the hypocone trait.

Conclusion

NMDTs showed a female predilection with bilateral predominance. A significant association was observed between these traits and dentoskeletal malocclusions. The most commonly observed NMDT was the presence of a hypocone on the upper second molars, followed by the tricuspid lower second premolars and the CoC.

## Introduction

During odontogenesis, disruption of differentiation of epithelial and mesenchymal tissues may result in aberrant manifestations of dental growth. The etiology of these developmental irregularities may stem from various factors, including local and systemic influences. Adult humans exhibit variations in the shape, size, and interproximal spaces of teeth. The morphological variation of these teeth within the oral cavity is distinct for each person, which serves as the foundation for identification and is used in forensic odontology [1]. Forensic odontology focuses mainly on the utilization of dental features and oral structures for identification within a legal framework [2]. The occurrence of dental anomalies can provide valuable insights into genetic and phylogenetic dimensions, given the involvement of over 300 genes in the complex mechanism of tooth development. Occasionally, these anomalies may stem from chromosomal irregularities, which could potentially exhibit X-linked patterns, consequently leading to varying prevalence rates between genders [3].

The morphological attributes of dental structures provide valuable insights for the analysis of human communities and play a fundamental role in categorizing human populations into taxonomic, phylogenetic, and evolutionary groupings. This capability stems from the widespread preservation of teeth, even under highly adverse conditions, in which skeletal remnants are discovered. Although the exclusive reliance on quantitative (metric traits) and qualitative (non-metric) characteristics of teeth may not conclusively establish a specific individual's identity, they can facilitate the process of personal identification by narrowing down potential ethnic and gender affiliations [4]. Non-metric dental traits (NMDTs) may be defined as either positive attributes (cusps) or negative features (pits, furrows, and grooves) that possess the potential to exist in a particular location (frequency), in varied forms or degrees (variability), and one or more individuals within a population group [5]. These findings establish the basis for the three-migration hypothesis [6] and illustrate the influence of dental anthropology on archaeology and related fields. These characteristics include the presence of Carabelli's cusp, hypocones in the upper second molars, molariform lower premolars, shoveling, and Bushman canines.

The Tubercle or cusp of Carabelli (CoC) is a prevalent dental morphological trait located on the mesiopalatal cusp of both maxillary deciduous second molars and maxillary permanent first molar crowns. This non-functional cusp exhibits a strong bilateral presence. Although clinically insignificant, it is important in the fields of forensic odontology and anthropology. Although inheritance is associated with an unknown etiology, it shows a genetic predisposition. The lack of provision for this cusp in orthodontic molar bands results in the accumulation of food particles in the space between the band and the tooth, potentially leading to the early onset of caries and periodontal diseases. Additionally, the Carabelli groove has been identified as an area susceptible to dental caries [7]. The hypocone is a cusp added to the primitive triangular upper molar teeth of therian mammals. Therefore, in mammals, the upper second molars are quadrilateral and resemble the first permanent molars. However, the distolingual cusp (hypocone) is sometimes reduced and the upper second molars have three prominent cusps, giving them a triangular shape. One study found that hypocone reduction was associated with the presence of CoC [8]. The lower second premolars show two occlusal patterns, three cusp or two cusp types, representing a ‘Y’-shaped groove pattern in three-cusp form, and ‘U,’ and ‘H’ shaped groove patterns in two-cusp form [9].

A significant association has been found between NMDTs and angle malocclusions with significant sex-linked differences [10]. Similarly, a significant association has been found between different malocclusions and different ABO blood groups. For example, Angle’s Class I malocclusion has been associated more with the O blood group, whereas Class II has been associated with the A blood group [11]. To date, no study has assessed the association between NMDTs and the type of dentoskeletal malocclusion and blood group in the Indian population. The primary objective of this study was to investigate the prevalence of NMDTs such as CoC in the permanent upper first molars, hypocone reduction in the upper second molar, and occlusal forms of the lower second premolar in the Maharashtrian population in both sexes. The secondary objective was to determine the association between NMDTs, malocclusion type, and ABO blood groups.

## Materials and methods

Study design

**Figure 1:**
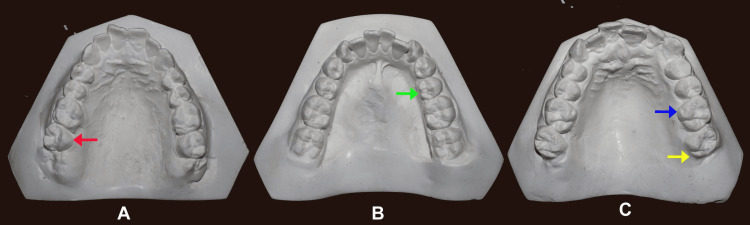
(A) Absence of hypocone on the upper second molar (red arrow), (B) Tricuspid lower second premolar (green arrow), and (C) Cusp of Carabelli on the upper first molar (blue arrow) and Hypocone on upper second molar (yellow arrow)

This prospective, observational, cross-sectional, single-center study was conducted in the Department of Oral Pathology in collaboration with the Department of Orthodontics on patients who attended the outpatient department (OPD) between September 2021 and September 2023. A convenience sampling method was adopted, where individuals were examined at baseline, and alginate impressions of the upper and lower arches were taken for the selected individuals. Various NMDTs such as CoC on the permanent upper first molar, hypocone (distolingual cusp in the upper second molar) presence on the upper second molar, and tricuspid form of the lower second premolars were noted (Figure 1).

The present study adhered to the guidelines outlined in the Strengthening the Reporting of Observational Studies in Epidemiology (STROBE) Statement. Approval for the study protocol was granted by the Institutional Ethical Committee, SMBT Dental College, Sangamner (EC/NEW/INST/2021/296), and all participants provided written informed consent as a component of a previous research endeavor conducted by the institution. This study was conducted in compliance with the ethical standards outlined in the Declaration of Helsinki. 

Sample size calculation

The sample size was calculated using https://www.calculator.net/ at an alpha error of 5% (95% confidence level) and a prevalence rate of the CoC in the Indian population of 87.60% [7]. The minimum sample size was determined as 174. Hence, to increase the power of the study to 95%, the present study was conducted on 528 individuals.

Study participants

A total of 528 individuals were selected based on the following inclusion criteria: healthy individuals aged 18-30 years whose past three generations were residents of Maharashtra; presence of all permanent teeth except third molars; absence of any congenital anomaly; or tooth-wearing diseases such as erosion, abrasion, and attrition affecting the teeth. Individuals with mixed parentage (paternal or fraternal) for the past three generations, fractured, decayed, carious, restored, or with a fixed prosthesis for the permanent upper first and second molars and lower second premolars were excluded from the study. The ABO blood groups of the patients were evaluated from their medical records. Detailed demographic data were obtained from all the participants.

Variables and data source

All individuals were further divided based on the type of dentoskeletal malocclusion as follows: Angle’s Class I molar relationship on the underlying Class I skeletal base as assessed by an ANB angle of 2-4 degree (ANB angle is the angle between points A and B, which are the deepest points in the anterior concavity of the maxilla and mandible, respectively, and the nasion point, which is the anterior most point on the frontonasal suture), Angle’s Class II molar relationship on the underlying Class II skeletal bases as assessed by an ANB angle of more than 4 degree, and Angle’s Class III molar relationship on the underlying Class III skeletal bases with an ANB angle of less than 2 degree. The dental relationship was assessed clinically on a dental chair, whereas the skeletal relationship was assessed using lateral cephalograms, which are routinely used as pretreatment diagnostic records of orthodontic patients. High-quality impressions of the upper and lower arches were obtained using alginate, and dental casts were carefully poured to avoid the entrapment of air bubbles. NMDTs, such as the CoC on the permanent upper first molar, hypocone on the upper second molar, and tricuspid form of the lower second premolars, were categorized into different patterns according to the dental anthropology system established by Arizona State University (ASUDAS) [11]. The ASUDAS framework for documenting NMDTs is rooted in the concept of visually representing the minimal and maximal expression of traits as well as the various degrees of expression between these two extremes. Although this approach can capture the most precise differentiation of any non-metric characteristic, these characteristics were categorized in the present study into two groups, namely present and absent traits, to facilitate and simplify the recording process.

Elimination of bias

To eliminate observer bias, all dental casts were coded and evaluated by two observers (an oral pathologist with more than five years of experience) under good lighting conditions, who were blinded to patient ID and sex. Dental and skeletal relationships were assessed by two experienced orthodontists. All measurements were repeated randomly in 50 individuals after two weeks by the same observers who were blinded to their previous scores. Statistical analysis was performed by another observer who was also blinded to patient ID and sex and was provided with an Excel sheet in the coded form. Only the principal investigator was aware of all the information.

Reliability

Intra- and inter-observer reliabilities were assessed in randomly selected 50 individuals at a two-week interval using the intra-class correlation coefficient (ICC). The intra- and inter-observer ICC values for NMDT measurements ranged from 0.88 to 0.92 and 0.84 to 0.90 respectively, for the skeletal relationship on lateral cephalogram ranged from 0.94 to 0.96 and 0.90 to 0.94 respectively, and for clinical assessment of the dental relationship ranged from 0.96 to 0.98 and 0.94 to 0.96 respectively indicating excellent reliability.

Statistical analysis

The data were processed utilizing IBM SPSS Statistics for Windows, Version 26 (Released 2019; IBM Corp., Armonk, New York, United States). Descriptive statistics included mean, standard deviation, frequency, and percentage. Data distribution normality was evaluated using the Shapiro-Wilk test. The chi-squared test of independence was used to assess the association between CoCs and other variables. The correlation between each measurement was determined using Spearman’s correlation test. Multivariate analysis enabled the identification of parameters that exhibited independent associations with NMDTs. A multinomial logistic regression model was constructed using NMDTs as the outcome variable. Instances of missing data points were excluded from the analysis. All analyses were performed at a 95% confidence level, with a significant difference of p<0.05.

## Results

The demographic data revealed that the study sample consisted of 134 (25%) males with a mean age of 20.82 ± 1.71 years and 394 (75%) females with a mean age of 21.15 ± 1.76 years. Class I dentoskeletal malocclusion was present in 145 (27%) patients, class II in 265 (50%), and class III in 118 (23%). This shows that class II dentoskeletal malocclusion was predominantly found in the Maharashtrian population, followed by classes I and III. A blood group was present in 132 (25%) individuals, B in 199 (38%) individuals, AB in 68 (13%) individuals, and O in 129 (24%) individuals, indicating that ABO blood group B was the most common blood group, followed by blood groups A, O, and AB, which was the least common blood group in the Maharashtrian population. The CoC was present in 184 (35%) individuals, hypocones in 365 (69%) individuals, and tricuspid lower second premolars in 224 (42%) individuals. NMDTs were most commonly observed in females with class II dentoskeletal malocclusion and in the B blood group. All NMDTs were observed predominantly in the B blood group (Table 1).

**Table 1:** Descriptive analysis of non-metric dental traits in the study population (n=528) SD: Standard deviation, Data presented in the form of N,%

Variables	Categories	Cusp of Carabelli (184, 34.84%)	Hypocone present (365, 69.12%)	Tricuspid lower second premolar (224, 42.42%)
Gender	Male (134, 25%)	56 (10.60%)	106 (20.07%)	62 (11.74%)
	Female (394, 75%)	128 (24.24%)	259 (49.05%)	162 (30.68%)
Dentoskeletal malocclusion	Class I (145, 27%)	28 (5.30%)	91 (17.23%)	28 (5.30%)
	Class II (265, 50%)	130 (24.62%)	184 (16.85%)	177 (33.52%)
	Class III (118, 23%)	26 (4.92%)	90 (34.84%)	19 (35.98%)
ABO blood group	A (132, 25%)	46 (8.71%)	85 (16.09%)	56 (10.60%)
	B (199, 38%)	70 (13.25%)	137 (25.94%)	89 (16.85%)
	AB (68, 13%)	21 (3.97%)	52 (9.84%)	27 (5.11%)
	O (129, 24%)	47 (8.90%)	91 (17.23%)	52 (9.84%)
Age (years)	Male (Mean ± SD)	20.82 ± 1.71		
	Female (Mean ± SD)	21.15 ± 1.76		

All NMDTs showed bilateral predominance (277 (76%) of hypocones, 119 (65%) of CoCs, and 91 (41%) of tricuspid lower second premolars). Both the hypocone and tricuspid lower second premolars showed left-sided predilection in 46 (12.5%) cases and 72 (32%) cases, respectively. In contrast, the CoC showed a right-sided predilection in 32(21%) cases. The presence of both hypocone and the CoC was not significantly associated with sex, type of dentoskeletal malocclusion, or ABO blood groups, whereas tricuspid lower second premolars were significantly associated with sex and type of dentoskeletal malocclusion (p<0.05) (Tables 2-4).

**Table 2:** Test of independence to assess association between presence of hypocone on both the sides and other variables *p value<0.05: Significant, Data presented in the form of N,%

Variables	Category	Hypocone N (%)			p-value	Effect size
		Bilateral 277 (76%)	Right 42 (11.5%)	Left 46 (12.5%)		
Gender	Male	87 (23.83%)	11 (3.08%)	9 (2.46%)	0.235	0.08
	Female	190 (52/05%)	31 (8.49%)	37 (10.13)		
ABO blood group	A	60 (16.43%	13 (3.56%)	13 (3.56%)	0.204	0.15
	B	107 (29.31%)	10 (2.73%)	18 (4.93%)		
	AB	38 (10.41%)	10 (2.73%)	5 (1.36%)		
	O	72 (19.72%)	9 (2.46%)	9 (2.46%)		
Dentoskeletal malocclusion	Class I	73 (20.00%)	14 (3.83%)	5 (1.36%)	0.075	0.15
	Class II	141 (38.63%)	16 (4.38%)	26 (7.12%)		
	Class III	63 (17.26%)	12 (3.28%)	15 (4.10%)		

**Table 3:** Test of independence to assess association between tricuspid lower second premolars on both the sides and other variables *p value<0.05: Significant, Data presented in the form of N,%

Variables	Category	Tricuspid lower second premolar N (%)			p-value	Effect size
		Bilateral 91 (41%)	Right 61 (27%)	Left 72 (32%)		
Gender	Male	11 (4.91%)	24 (10.71%)	27 (12.05%)	0.0001*	0.29
	Female	80 (3.57%)	37 (16.51%)	45 (20.08%)		
ABO blood group	A	18 (8.03%)	16 (7.14%)	23 (10.26%)	0.385	0.17
	B	40 (17.85%)	24 (10.71%)	24 (10.71%)		
	AB	11 (4.91%)	10 (4.46%)	6 (2.67%)		
	O	22 (9.82%)	11 (4.91%)	19 (8.48%)		
Dentoskeletal malocclusion	Class I	10 (4.46%)	6 (2.67%)	12 (5.35%)	0.049*	0.21
	Class II	75 (33.48%)	53 (23.66%)	49 (21.87%)		
	Class III	6 (2.67%)	2 (00.89%)	11 (4.91%)		

**Table 4:** Test of independence to assess association between Cusp of Carabelli on both the sides and other variables *p value<0.05: Significant, Data presented in the form of N,%

Variables	Category	Cusp of Carabelli N (% )			p-value	Effect size
		Bilateral 119 (65%)	Right 38 (21%)	Left 27 (14%)		
Gender	Male	39 (21%)	11 (6%)	6 (3%)	0.546	0.08
	Female	80 (43%)	27 (15%)	21 (11%)		
ABO blood group	A	28 (15%)	13 (7%)	5 (3%)	0.529	0.17
	B	45 (24%)	12 (7%)	13 (7%)		
	AB	12 (7%)	6 (3%)	3 (2%)		
	O	34 (18%)	7 (4%)	6 (3%)		
Dentoskeletal malocclusion	Class I	20 (11%)	4 (2%)	4 (2%)	0.227	0.18
	Class II	83 (45%)	31 (17%)	16 (9%)		
	Class III	16 (9%)	3 (2%)	7 (4%)		

There was no significant association between the CoC and gender (p =0.056), CoC, or ABO (p =0.891). A statistically significant association was found between CoC, dentoskeletal malocclusion, hypocone, and tricuspid lower second premolars (p <0.05). Both CoC and hypocone were present (4-cusp maxillary second molars) in 142 (27%) individuals, whereas the CoC and hypocone reduction (3-cusp maxillary second molars) were present in 42 (8%) individuals. Similarly, CoCs with tricuspid lower second premolars were present in 90 (17%) patients, whereas CoCs with bi-cuspid lower second premolars were present in 94 (18%) patients. The CoC was significantly associated with dentoskeletal malocclusion (p=.001), predominantly with class II relationships in 130 cases (25%), as shown in Table 5.

**Table 5:** Test of independence to assess the association between the Cusp of Carabelli and other variables *p value<0.05: Significant, Data presented in the form of N,%

Variables	Category	Cusp of Carabelli N(%)		p-value	Effect size
		Yes 184 (35%)	No 344 (65%)		
Gender	Male	56 (11%)	78 (15%)	0.056	0.08
	Female	128 (24%)	266 (50%)		
ABO blood group	A	46 (9%)	86 (16%)	0.891	0.03
	B	70 (13%)	129 (24%)		
	AB	21 (4%)	47 (9%)		
	O	47 (9%)	82 (16%)		
Hypocone in upper second molar	Present (4-cusp)	142 (27%)	223 (42%)	0.003*	0.13
	Absent (3-cusp)	42 (8%)	121 (23%)		
Tricuspid lower second premolar	Present (3-cusp)	90 (17%)	134 (25%)	0.027*	0.09
	Absent (2-cusp)	94 (18%)	210 (40%)		
Dentoskeletal malocclusion	Class I	28 (5%)	117 (22%)	0.001*	0.3
	Class II	130 (25%)	135 (26%)		
	Class III	26 (5%)	92 (17%)		

All NMDTs showed a negative correlation with sex, although the correlation was not statistically significant, indicating that all the observed traits showed female predominance. There was a positive correlation between age and the presence of hypocones in the upper second molars and CoC in the upper first molars, which showed that both traits were more prevalent in older age groups. This correlation was statistically significant for the CoC (p=.024). The tricuspid lower second premolars showed a negative correlation with age, indicating that they were more prevalent in the younger age group. All age groups showed a positive correlation with dentoskeletal malocclusion, which was statistically significant for the presence of hypocones in the upper second molars, indicating that the presence of NMDTs increased with an increase in malocclusion. There was a weak positive correlation between the ABO blood group and all NMDTs; however, this correlation was not statistically significant. There was a statistically significant positive correlation between the presence of CoCs, hypocones, and tricuspid lower second premolars (p <0.05) (Table 6).

**Table 6:** Spearman correlation between variables *p value<0.05: Significant

Variable		Cusp of Carabelli	Dentoskeletal malocclusion	Gender	Presence of hypocone	ABO blood group	Tri-cuspid lower second premolar	Age
Cusp of Carabelli	r value	—						
	p value	—						
Dentoskeletal malocclusion	r value	0.049	—					
	p value	0.261	—					
Gender	r value	-0.085	0.045	—				
	p value	0.051	0.3	—				
Presence of hypocone	r value	0.127	0.103	-0.126	—			
	p value	0.003*	0.018*	0.004*	—			
ABO blood group	r value	0.002	0.047	0.005	0.057	—		
	p value	0.972	0.282	0.91	0.194	—		
Tricuspid lower second premolar	r value	0.096	0.025	-0.045	0.051	-0.025	—	
	p value	0.027*	0.056	0.298	0.242	0.566	—	
Age	r value	0.098	-0.051	-0.178	0.026	-0.049	-0.046	—
	p value	0.024*	0.239	.001*	0.55	0.264	0.295	—

Multinomial logistic regression model analysis revealed that none of the independent variables were statistically significant predictors of the presence of CoC and tricuspid lower second premolars, while dentoskeletal malocclusion and sex were significant predictors of the presence of the hypocone trait (Table 7). The multinomial logistic regression analysis of dependent variables (CoC, hypocone and tricuspid premolar) with independent variables (blood group, malocclusion, age) to predict the occurrence was calculated by following formula: CoC in the upper first molar (t = -3.1149 + 0.01169 Blood group + 0.1370 Malocclusion + 0.1060 Age), Hypocone in the upper second molar (t = 1.0164 - 0.09880 Blood group - 0.3182 Malocclusion - 0.04754 Age) and Tricuspid lower second premolar (t = -1.1827 + 0.05577 Blood group - 0.03504 Malocclusion + 0.06906 Age); where t stands for value of dependent variable. The scoring criteria was as follows: blood group (1-A,2-B,3-AB and 4-O) and malocclusion (1-Class1, 2-Class2 and 3-Class3).

**Table 7:** Multinomial logistic regression model *p value<0.05: Significant

Variable	Factors	Age	ABO blood group	Dentoskeletal malocclusion	Gender	Probability
Cusp of Carabelli	Coefficient	0.056	0.007	0.094	-0.216	0.095
	P value	0.084	0.887	0.251	0.094	
	Odd ratio	1.095	1.011	1.157	0.704	
Hypocone in the upper second molar	Coefficient	0.01	0.06	0.203	-0.416	0.002*
	P value	0.748	0.249	0.014*	0.003*	
	Odd ratio	1.019	1.103	1.404	0.495	
Tricuspid lower second premolar	Coefficient	-0.05	-0.035	0.025	-0.168	0.397
	P value	0.118	0.482	0.744	0.189	
	Odd ratio	0.922	0.945	1.041	0.763	

## Discussion

Analysis of NMDTs yields valuable insights into how minor evolutionary changes contribute to racial variation and possess important taxonomic and forensic significance. This importance is more pronounced when there is a need to guide the classification of individuals based on potential gender and race using unidentified remains. Several archaeological studies have noted similarities in the occurrence and pattern of NMDTs among different ethnic populations in determining ancestry in forensic dental anthropology. This scenario can be achieved with the availability of population-specific data on the prevalence of NMDTs [12]. The objective of this study was to establish the prevalence rates of various NMDTs within the Maharashtrian population and the association between the occurrence of these traits and other variables, such as dentoskeletal malocclusion and ABO blood group. To the best of our knowledge, this is the first study to assess such associations in CoCs, tricuspid lower second premolars, and hypocone presence.

The results of the present study revealed that all NMDTs were seen predominantly in females, which is in accordance with previous studies [13,14] and in contrast with the findings of Ramesh et al. [15]. These contradictory results might be because the authors conducted the study in a North Indian population, and ethnic differences have been documented in the presence of NMDTs [3]. CoCs in both the upper first molars and hypocones in the upper second molars were present in older individuals, whereas tricuspid lower second molars were more prevalent in younger individuals. This finding is in agreement with a study by Jain et al., who found a higher prevalence of NMDTs in the age group of less than 25 years [16].

The present study revealed that Class II dentoskeletal malocclusion was significantly associated with the presence of NMDTs, particularly hypocones in the upper second molars. Similar findings were reported by Ashoori et al. [17], and contrasting findings were reported by Fernandez et al. [18], who reported a higher prevalence of Class III malocclusion. They conducted a study of the Brazilian population, which may have resulted in contradictory findings. Our study indicated a weak positive correlation between the ABO blood group and NMDTs. A higher prevalence of NMDTs was observed in the B blood group, followed by the O and A blood groups. As no study has been conducted to relate the presence of NDMTs to blood groups, this finding could not be compared with previous literature. Previous studies have reported an association between the type of malocclusion and the blood group. A higher prevalence of Class II malocclusions has been observed in the O and B blood groups [19]. As in our study, NMDTS has been associated with Class II malocclusion; therefore, it might be associated with a higher prevalence of B and O blood groups, as shown in previous studies [19,20]. The regional distribution of ABO blood groups varies across populations. The pathogenesis of malocclusion is complex and multifaceted, and its precise etiology remains elusive, although genetic components are likely to be influential.

A strong association was observed between the prevalence of the CoC in the upper first molars and the hypocone in the upper second molars. This might be due to genetic linkages. It has been noticed A previous study showed that the X chromosome plays a significant role in determining the crown size of the upper first and second molars. This is due to the impact of karyotypes containing different numbers of active gene regions on the development of tooth germs during the initial phase of human odontogenesis. Subsequent formation of cusps, such as the hypocone and CoC, could potentially be influenced by this fundamental developmental process [21]. The presence of this linkage has been predominantly identified among females, a factor that could explain the elevated prevalence of NMDTs in the female cohort examined in this study. Additionally, this association was linked to blood groups, suggesting a genetic predisposition to these traits.

The patterning cascade model (PCM) considers variations in the size and shape of the molar crown. According to the PCM hypothesis, in typical populations, the position of the cusps that develop early (upper first molar) influences the growth of additional cusps that develop later (upper second molar). When the early cusps are positioned closer to each other within a relatively larger tooth germ, creating space for the later-forming cusp, it is expected that the later cusps will be present and larger in outer free space. In populations without abnormalities, the decreased distance between cusps on the maxillary permanent molars plays a significant role in promoting CoC expression. Specifically, the reduced distance between the mesiobuccal and mesiolingual cusps is considered a critical factor in the expression of the CoC. This indicates that the positioning of cusps within the tooth germ can influence the development and size of additional cusps as the tooth matures. The PCM hypothesis sheds light on the intricate relationship between cusp development and tooth crown size and shape in a normal population. Understanding these patterns can provide valuable insights into the formation of dental features and variations in molar morphology in the human population. Further research into the PCM hypothesis may uncover additional details regarding the mechanisms underlying cusp development and their implications in dental anthropology and evolutionary biology [22].

Based on an analysis of research conducted on the dentinoenamel junction, it has been observed that the distolingual cusp and Carabelli’s cusp originate from identical lingual cingulum areas within typical populations [23]. In the context of the upper first permanent molars, a standard configuration featuring four cusps is widely prevalent, whereas a three-cusp arrangement is frequently identified in the second permanent molars. These findings underscore the consistent anatomical variations in tooth morphology among different populations, shedding light on the diverse patterns of cusp development in human dentition. The expression of the extra cusp (CoC) is significantly associated with the expression of the extra cusp (hypocone) in upper second molars [21].

Similar reasons might also be associated with the presence of extra cusps on the lower second premolars (tricuspid lower second premolars), as observed in the present study. Moreover, the second premolar serves as an intermediary structure bridging the gap between the premolar and first molar, typically characterized by the presence of one or two lingual cusps that aid in the efficient chewing process when in contact with the corresponding teeth in the opposing dental arch. This dental feature plays a crucial role in the overall functionality and effectiveness of the masticatory system [24].

The most common NMDT observed in the present study was the presence of a hypocone on the upper second molars, followed by the tricuspid lower second premolars, and CoC on the upper first molars. The key strength of the present study is that no previous study has been done to assess the association of the NMDTs with both malocclusion and ABO blood groups. This finding could not be compared, as no study has been conducted on the Maharashtrian population regarding the presence of NMDTs, and it has been well documented in the literature that racial and ethnic differences exist in the appearance of these traits; therefore, they show different prevalence rates in different populations [3,25].

Limitations

The primary constraint lies in the restricted sample size, which hinders the thorough examination of any potential genetic connections with the identified anomaly. Furthermore, the absence of observations regarding the genetic link poses a significant limitation to the comprehensive analysis of the anomaly in question. To address this limitation effectively, future research endeavors could expand the scope of the study by including larger sample sizes and diverse populations, thereby enabling a more robust and generalized understanding of the association between NMDTs and dentoskeletal malocclusion and ABO blood groups.

## Conclusions

The results of the present study indicated that non-metric dental traits were predominantly found in females with Class II dentoskeletal malocclusion and the B blood group, with bilateral predilection. The most commonly observed NMDT was the presence of a hypocone on the upper second molars, followed by the tricuspid lower second premolars and the CoC. Dentoskeletal malocclusion and sex are significant predictors of hypocone traits. A statistically significant association was found between all traits and dentoskeletal malocclusion.
